# Child malnutrition in Ifanadiana district, Madagascar: associated factors and timing of growth faltering ahead of a health system strengthening intervention

**DOI:** 10.1080/16549716.2018.1452357

**Published:** 2018-03-29

**Authors:** Sarah McCuskee, Andres Garchitorena, Ann C. Miller, Lara Hall, Mohammed Ali Ouenzar, Victor R. Rabeza, Ranto H. Ramananjato, Hery-Tiana Rahaniraka Razanadrakato, Marius Randriamanambintsoa, Michele Barry, Matthew H. Bonds

**Affiliations:** a Stanford University School of Medicine, Stanford, CA, USA; b Harvard Medical School, Department of Global Health and Social Medicine, Boston, MA, USA; c PIVOT, Ranomafana, Madagascar; d Division of Global Health Equity, Brigham and Women’s Hospital, Boston, MA, USA; e Institut National de la Statistique, Direction de la Demographie et de les Statistiques Sociales, Antananarivo, Madagascar; f UNICEF, Madagascar Country Office, Antananarivo, Madagascar; g Center for Innovation in Global Health, Stanford University, Stanford, CA, USA; h Office of the Dean, Stanford University School of Medicine, Stanford, CA, USA

**Keywords:** Child undernutrition, stunting, wasting, chronic malnutrition, acute malnutrition, household survey

## Abstract

**Background**: Child malnutrition, a leading cause of death and disability worldwide, is particularly severe in Madagascar, where 47% of children under 5 years are stunted (low height-for-age) and 8% are wasted (low weight-for-height). Widespread poverty and a weak health system have hindered attempts to implement life-saving malnutrition interventions in Madagascar during critical periods for growth faltering.

**Objective**: This study aimed to shed light on the most important factors associated with child malnutrition, both acute and chronic, and the timing of growth faltering, in Ifanadiana, a rural district of Madagascar.

**Methods**: We analyzed data from a 2014 district-representative cluster household survey, which had information on 1175 children ages 6 months to 5 years. We studied the effect of child health, birth history, maternal and paternal health and education, and household wealth and sanitation on child nutritional status. Variables associated with stunting and wasting were modeled separately in multivariate logistic regressions. Growth faltering was modeled by age range. All analyses were survey-adjusted.

**Results**: Stunting was associated with increasing child age (OR = 1.03 (95%CI 1.02–1.04) for each additional month), very small birth size (OR = 2.32 (1.24–4.32)), low maternal weight (OR = 0.94 (0.91–0.97) for each kilogram, kg) and height (OR = 0.95 (0.92–0.99) for each centimeter), and low paternal height (OR = 0.95 (0.92–0.98)). Wasting was associated with younger child age (OR = 0.98 (0.97–0.99)), very small birth size (OR = 2.48 (1.23–4.99)), and low maternal BMI (OR = 0.84 (0.75–0.94) for each kg/m^2^). Height-for-age faltered rapidly before 24 months, then slowly until age 5 years, whereas weight-for-height faltered rapidly before 12 months, then recovered gradually until age 5 years but did not reach the median.

**Conclusion**: Intergenerational transmission of growth faltering and early life exposures may be important determinants of malnutrition in Ifanadiana. Timing of growth faltering, in the first 1000 days, is similar to international populations; however, child growth does not recover to the median.

## Background

Malnutrition, here meaning undernutrition, is a leading cause of death and disability worldwide [1], and contributes significantly to mortality in individuals affected by other diseases, particularly children with diarrhea, pneumonia, and measles [2]. Acute malnutrition presents as wasting (low weight for height) [3] and is responsible for an estimated 13% of child deaths globally [4]. Chronic malnutrition tends to slow or decrease linear growth, presents as stunting (low height for age), and is responsible for an estimated 14% of child deaths [4]. Nutrition in the first 1000 days (gestation and the first two years of life) is particularly important in determining health and nutritional status in later childhood and adult life [5]. Among malnourished children, linear growth (height-for-age) can falter rapidly until age 2 years, then slowly thereafter, whereas weight-for-height, which exhibits more variability, falters until 1 year of age, then tends to recover to international medians [6]. Faltering in either linear growth or weight gain by age 2 predicts shorter adult height and lower educational attainment, and malnutrition in the first year of life predicts low socioeconomic status in adulthood [5,7,8].

Managing acute malnutrition using WHO guidelines has the potential to save more lives globally than any other existing nutrition intervention; it also could substantially decrease the prevalence of wasting and stunting by allowing child growth to recover to international norms [9]. Inpatient and outpatient treatment of severe and moderate acute malnutrition is part of the WHO Essential Nutrition Actions (ENAs) [10,11]. Other ENAs, in order of effectiveness, include: breastfeeding, complementary feeding, supplementation for mothers and children, and fortification. Combined, these ENAs are well studied, cost effective and could save an estimated 1 million lives if scaled to 90% coverage worldwide [9].

Comparatively little is known about the etiology of malnutrition, which might help target these or other interventions to prevent it. A wide array of risk factors have been studied, lending support to nutrition-specific interventions in the context of health system strengthening; however the importance of these risk factors varies widely between studies and environments [4]. Diarrheal illness has been classically associated with malnutrition in general [12], but in a recent systematic review, this was true more frequently for wasting than for stunting [13]. The association of diarrheal disease with wasting is supported by cross-sectional evidence from Ghana, an association exacerbated by lack of toileting facilities [14]. In another study, community-level sanitation access protected from stunting regardless of household-level sanitation access [15], however poor sanitation at the household level also predicted stunting, supporting the importance of diarrheal illness and poor sanitation in malnutrition [15]. Gastrointestinal infection, even without symptoms such as diarrhea, is likely to play a role in stunting as well: a recent longitudinal study attributed linear growth faltering, which leads to stunting, to enteropathogen exposure, even in the absence of diarrheal illness [16]. Diet is also important, although it is less commonly associated with stunting or wasting in international literature, perhaps due to difficulties in studying nutritional intake [17]. However, some longitudinal research attributes both linear growth faltering and poor weight gain to low energy intake and low protein density [16]. Environmental factors such as rural residence [13,17], dry season, and local food shortages [18] all predict malnutrition in multiple studies. Maternal and paternal education have been associated with lower odds of malnutrition [13,19]; in one study in Indonesia, health-promoting parental behaviors such as vaccination and supplementation appeared to mediate this relationship [20], and in one study in Tanzania, maternal farming work appeared to worsen child nutritional status [18]. However across international literature, the strongest and most consistent risk factors for malnutrition are demographic: male sex [13,17,21], increasing child age [13,14,21], smaller birth size [13,14,19], and poor maternal nutritional status [13,14,17,19] all predicted malnutrition, particularly stunting, in multiple studies and reviews, leading some authors to conclude that many relevant determinants of growth faltering are already established at birth [19].

Madagascar is one of the world’s poorest countries with the lowest per capita spending on health care [22]. Food insecurity, high infectious disease prevalence, and climate shocks make Madagascar’s population particularly vulnerable to both acute and chronic malnutrition [23]. In 2013, stunting prevalence was 47.3% and the wasting prevalence was 8.2% [24]. The etiology of malnutrition in young Malagasy children has been variously attributed to behavioral factors, food availability, maternal education, male sex, and infectious disease, but data are sparse and most studies are small [25–29]. Given the wide array of factors associated with malnutrition in the international literature, targeted research investigating risk factors for malnutrition in Madagascar can contribute to a consensus which might further inform public health action.

Although official government plans for public health action and implementation of ENAs have been in place since 2004 in Madagascar, weak health systems marked by political instability, resource limitations, and institutional fragmentation have limited their progress [23]. Implementation has largely relied on the efforts of Malagasy NGOs in their local areas, with support from the Ministry of Health and Office National de la Nutrition (ONN), and is often vertically oriented, with nutrition managed through separate protocols from other child, maternal, and public health initiatives. Other efforts to combat and prevent chronic malnutrition have been implemented in the country. International aid projects have included semi-annual nutrition screening and vaccination days, behavior change communication, mass media initiatives, and Integrated Management of Childhood Illness (IMCI) implementation at local health centers [30,31]. Local NGO efforts have supported a community health worker program in which locally elected, paid community agents (Agent Communitaire, AC) monitor child weight and provide nutrition and breastfeeding advice to mothers, similar to child health programs in other countries [27]. One World Bank-supported randomized controlled trial is currently testing the effects of supplementation and parenting education [26]. These initiatives have not generally been directed toward the management of acute malnutrition, and have not addressed, or adapted to, the etiology of malnutrition in Madagascar.

As part of a broader health system strengthening intervention, the non-governmental health organization PIVOT partnered with the Ministry of Health (MoH) of Madagascar [32] to support screening and treatment of acute malnutrition in the rural district of Ifanadiana, starting in 2015. Prior to the intervention, a district-wide population survey was carried out among 1522 households to obtain robust estimates of the health and socio-economic conditions of the population of Ifanadiana, including prevalence of malnutrition in children [33]. A detailed understanding of the main determinants of both chronic and acute malnutrition in this population could help inform local strategies against malnutrition in Ifanadiana and will contribute to the scientific understanding of child malnutrition in low-resource settings.

## Methods

### Study setting

Ifanadiana is a rural health district located in the southeast of the country. The population of Ifanadiana comprises approximately 148,000 residents, living in 13 communes and 195 fokontany (village areas). Ifanadiana’s health system consists of one district hospital (CHD) and at least one basic health center (CSB 2) per commune that provides primary care and supervises a system of community health workers (ACs), two per fokontany; six communes also have a second, more limited health center (CSB 1). There is one district hospital (CHD) with approximately 40 beds.

The 2014 district-wide survey conducted in Ifanadiana revealed that stunting and wasting prevalences were similar to national figures (at 51.3% and 10.2%, respectively) and child mortality was twice as high as for Madagascar as a whole, at 145 per 1000 live births [33]. The primary economic activity in Ifanadiana is subsistence agriculture, and more than 70% of households lived in extreme poverty [33]. PIVOT’s malnutrition program began in 2015, providing screening, treatment of acute malnutrition at the health center and hospital levels, and included home follow-up at the community level.

### Study design

This study consists of a secondary analysis of data from this cross-sectional household survey carried out by the National Institute of Statistics (INSTAT) in 2014, which constituted the baseline of a longitudinal cohort measuring population-level health impacts of the health system strengthening intervention implemented in Ifanadiana. The baseline survey methods and results, including sampling and data processing, have been published in detail [33,34].

### Study sampling

The baseline survey covered the entire district of Ifanadiana, and was a population-representative, two stage cluster sampling design in two strata (PIVOT initial catchment area and non-catchment area; these are relevant for the planned longitudinal cohort study but not for this analysis), including 80 clusters and 1600 households.

### Study population and data collection

Survey content was based on the Madagascar DHS, with additional questions on child development from the Multiple Indicator Cluster Survey (MICS), questions on adult health from Rwanda’s Questionnaire de Bien-Être, and household economic data from the World Bank Living Standard Measurement Survey.

Data collection was conducted between April and May 2014 through face-to-face interviews. Teams of four data collectors and one field supervisor, professionally trained by INSTAT, visited each of the 1600 sampled households; all households sampled were selected. In each selected household, basic demographic and health data were collected for all *de facto* residents (including everyone normally present and everyone who had spent the night prior in the household). Interviews and anthropometric measurements were taken for all children 5 years of age and under, and all adults of reproductive age, defined as women ages 15–49 and men ages 15–59. Anthropometry consisted of measuring weight and height (or length for infants). Children’s weights and lengths or heights were recorded as the average of three measurements. Weights were collected using Seca 876 electronic medical scales and lengths or heights were measured in centimeters using portable adult/pediatric measuring boards. Age and *de facto* household residence were the only inclusion criteria for household members; no members were otherwise excluded. Data were then double-entered in CSPro and internal consistency was assessed.

### Ethics

The study was approved by the Madagascar National Ethics Committee, INSTAT, and the Harvard Medical School Institutional Review Board (IRB). This malnutrition-focused analysis was approved by the Stanford School of Medicine IRB. All data analyzed had individual identifiers removed.

### Data analysis

Sections of the survey relevant to this analysis included household members’ age, sex, education, recent illnesses and care-seeking behavior, child health and development, child and maternal mortality, women’s and men’s health, household sanitation, including drinking water source and toilet facilities, and wealth. As is standard in DHS surveys, the wealth score was constructed from principal component analysis of household assets at the time of interview [33] and provides an overall indication of the socioeconomic status of the household. Birth size relative to peers is a variable reported by each child’s mother, indicating whether the child was larger than average, smaller than average, or average compared to other children at birth. Vaccination and supplementation data were based on whether a child had ever received the vaccine or supplement in question; age at administration may vary slightly between children because many vaccinations and supplements are administered at large-scale health fairs. A full list of variables is provided in Table 1.

**Table 1.** Descriptive statistics for all variables included in the analysis, adjusted for survey characteristics.

Continuous variables	Mean (SE)	N	Categorical variables	Proportion (SE)	N
**Child**					
HAZ	−2.04 (0.06)	1175	Sex (Proportion female)	0.50 (0.02)	1175
WHZ	−0.94 (0.05)	1175	Birth size relative to peers: very small	0.11 (0.01)	1175
Child age (mo)	33.5 (0.50)	1175	Smaller than average	0.22 (0.02)	
Proportion of childhood vaccines received	0.75 (0.01)	906	Average	0.27 (0.02)	
Birth rank	4.03 (0.12)	1175	Larger than average	0.26 (0.01)	
Birth space from previous child (mo)	44.5 (1.98)	936	Very large	0.13 (0.01)	
			Mother alive	1.00 (0)	1175
			Live with mother	1.00 (0.0006)	1175
			Breastfed	1.00 (0.002)	1175
			Singleton birth	0.98 (0.005)	1175
			Measles vaccinated	0.73 (0.02)	977
			BCG vaccinated	0.63 (0.03)	977
			Vitamin A administered	0.53 (0.02)	1175
			Iron administered	0.04 (0.01)	1157
			Deworming administered	0.73 (0.02)	1175
			Doses of polio vaccine: 0–2	0.24 (0.02)	925
			3 (complete)	0.57 (0.03)	
			4	0.18 (0.03)	
			5+	0.01 (0.004)	
			Doses of DTaP vaccine: 0–2	0.25 (0.02)	930
			3 (complete)	0.73 (0.02)	
			4	0.01 (0.004)	
			5	0.01 (0.003)	
			6+	0.00 (0.0003)	
			Ill or injured in past 4 weeks	0.69 (0.02)	1174
**Mother**					
Maternal BMI	19.9 (0.13)	1170	Mother works outside home	0.99 (0.005)	1175
Maternal weight (kg)	44.6 (0.33)	1170	Mother literate	0.63 (0.03)	752
Maternal height (cm)	150 (0.22)	1170			
Maternal age (y)	28.4 (0.26)	1175			
Mother’s education (years)	1.99 (0.16)	1175			
Mother’s parity	4.43 (0.13)	1175			
					
					
**Father**					
Paternal BMI	19.7 (0.11)	849	Father works outside home	1.00 (0)	849
Paternal weight (kg)	50.7 (0.39)	850	Father literate	0.66 (0.03)	617
Paternal height (cm)	160 (0.31)	849	Father smokes	0.14 (0.02)	851
Paternal age (y)	33.3 (0.42)	851	Father has health insurance	0.03 (0.01)	851
Paternal education (y)	2.34 (0.18)	851			
Father’s alcohol use (drinks/past 24 h)	1.88 (0.22)	440			
**Household**					
Household wealth score (log10)	−0.15 (0.02)	1175	Water disinfected	0.27 (0.02)	1175
Child deaths in household	0.90 (0.06)	1175	Water source: Surface water (river, lake)	0.45 (0.04)	1175
Household size	6.56 (0.12)	1175	Unprotected well	0.03 (0.01)	
Proportion of household ill in past 4 weeks	0.50 (0.01)	1154	Protected well	0.01 (0.003)	
			Tap in the street	0.00 (0.002)	
			Tap in the house	0.00 (0.002)	
			Public fountain/tap	0.09 (0.03)	
			Unprotected spring	0.40 (0.03)	
			Protected spring	0.02 (0.01)	
			Toilet: Flush toilet connected to pit latrine	0.00 (0.0009)	1175
			Flush toilet connected to septic tank	0.01 (0.004)	
			Ventilated improved pit latrine	0.00 (0.002)	
			Pit latrine with slab	0.00 (0.003)	
			Pit latrine without slab/open pit	0.41 (0.04)	
			No facility	0.58 (0.04)

**Table 2.** Logistic regression models predicting stunting or wasting. Univariate odds ratios and final model odds ratios shown for each model. Wald test <0.1 for all variables in final models. Adjusted for survey characteristics.

	Stunting Model A1 (N = 1170)			Stunting Model B1 (N = 845)			Wasting Model A2 (N = 1170)		
		F 9.86			F 9.61			F 6.39	
		Prob > F 2.30 × 10^–9^			Prob > F 1.00 × 10^–9^			Prob > F 1.66 × 10^–5^	
		Hosmer-Lemeshow p 0.53			Hosmer-Lemeshow p 0.82			Hosmer-Lemeshow p 0.40	
	Univariate	Multivariate		Univariate	Multivariate		Univariate	Multivariate	
	OR (95%CI)	OR (95%CI)	p	OR (95%CI)	OR (95%CI)	p	OR (95%CI)	OR (95%CI)	p
**Child**									
Child age (mo)	1.02 (1.01–1.03)	1.03 (1.02–1.04)	<0.001	1.02 (1.01–1.03)	1.03 (1.02–1.04)	<0.001	0.98 (0.97–0.99)	0.98 (0.97–0.99)	0.008
Birth size relative to peers: very small	2.46 (1.46–4.16)	2.34 (1.35–4.06)	0.003	2.46 (1.46–4.16)	2.32 (1.24–4.32)	0.009	2.54 (1.31–4.94)	2.48 (1.23–4.99)	0.012
Smaller than average	1.93 (1.25–2.98)	1.66 (1.10–2.52)	0.017	1.93 (1.25–2.98)	1.87 (1.14–3.09)	0.014	1.84 (1.07–3.17)	1.69 (1.00–2.88)	0.051
Average	1 (ref)	1 (ref)		1 (ref)	1 (ref)		1 (ref)	1 (ref)	
Larger than average	1.32 (0.88–1.98)	1.33 (0.90–1.97)	0.140	1.32 (0.88–1.98)	1.33 (0.82–2.17)	0.243	1.23 (0.71–2.12)	1.22 (0.69–2.16)	0.487
Very large	1.18 (0.67–2.06)	1.18 (0.67–2.10)	0.560	1.18 (0.67–2.06)	1.46 (0.81–2.62)	0.200	0.80 (0.30–2.14)	0.87 (0.31–2.39)	0.780
Deworming administered	1.32 (1.00–1.73)			1.32 (1.00–1.73)					
Vitamin A administered							0.59 (0.37–0.94)		
**Mother**									
Maternal weight (kg)	0.92 (0.90–0.95)	0.93 (0.90–0.96)	<0.001	0.92 (0.90–0.95)	0.94 (0.91–0.97)	<0.001			
Maternal height (cm)	0.92 (0.90–0.95)	0.96 (0.92–0.99)	0.010	0.92 (0.90–0.95)	0.95 (0.92–0.99)	0.028			
Maternal BMI (kg/m^2^)							0.82 (0.73–0.92)	0.84 (0.75–0.94)	0.002
Maternal parity	1.04 (1.00–1.10)			1.04 (1.00–1.10)					
Maternal education (y)							0.88 (0.78–0.99)		
**Father**									
Paternal weight (kg)				0.95 (0.91–0.98)					
Paternal height (cm)				0.94 (0.91–0.96)	0.95 (0.92–0.98)	<0.001			
Paternal education (y)				0.95 (0.90–1.01)					
**Household**									
Wealth score (log10)	0.79 (0.50–1.24)	1.70 (0.97–3.00)	0.064	0.79 (0.50–1.24)	1.94 (0.96–3.93)	0.064	0.33 (0.16–0.67)		
Child deaths in household	1.11 (0.98–1.25)			1.11 (0.98–1.25)					
Proportion of household ill in last 2 weeks							0.52 (0.23–1.17)		
Water disinfected	0.72 (0.52–0.98)			0.72 (0.52–0.98)					
Water source: Surface water (river, lake)							1 (ref)		
Unprotected well							0.42 (0.09–1.91)		
Protected well							-		
Tap in the street							-		
Tap in the house							-		
Public fountain/tap							0.53 (0.20–1.43)		
Unprotected spring							0.92 (0.60–1.40)		
Protected spring							-		

Anthropometric measurements and age were used to construct dummy variables reflecting child stunting (low height for age) or wasting (low weight for height), using internationally recognized z-scores and thresholds [3]. We defined stunting as height for age z-score (HAZ) <−2. Similarly, we defined wasting as weight for height z-score (WHZ) <−2. Descriptive statistics (means and proportions for continuous and categorical variables, respectively) were calculated. Univariate analysis was conducted comparing stunted to non-stunted and wasted to non-wasted children for exposure variables including child health and anthropometry, perinatal care, vaccination, maternal and paternal health and anthropometry, and household water, sanitation, and wealth.

Stunting and wasting were used as outcome variables in separate logistic regression models, using the same methods for each. Variables associated (p < 0.10) with stunting/wasting in univariate analysis (chi-squared or t-tests for categorical and continuous data, respectively) were examined for normality visually using histograms. Variables with insufficient heterogeneity (≥95% same response) were not tested. Wealth score was found to have positive skew and was transformed using base 10 logarithm. These variables were then added to multiple logistic regression models describing predictors of stunting/wasting in two subpopulations, A (all children, N = 1175) and B (those children with complete paternal data, N = 845). Stepwise model selection was performed through backwards elimination based on predictor p < 0.05. Final models were reached when p < 0.05 for all predictors. Goodness of fit was evaluated using the Hosmer-Lemeshow test, and individual variables were evaluated using Wald tests. All models were tested with and without wealth score included, and the better-fitting model was selected. Variance inflation factors were calculated, along with covariance matrices, to assess multicollinearity in all final models. All analyses were performed in Stata 13 using survey adjustment with appropriate sampling weights and Taylor linearized variance estimation.

## Results

Survey response rate was 95.5% (1522 households surveyed of 1600 sampled). Among respondents, we gathered anthropometric data for 1175 children from 6 months to 5 years old, as well as for their parents when present. All eligible children were included in the analysis. Complete data were available for almost all child-, maternal-, and household-level variables, while fewer data were available for fathers (N = 845) as not all children’s fathers were in the household at the time of the survey.

**Figure 1.**
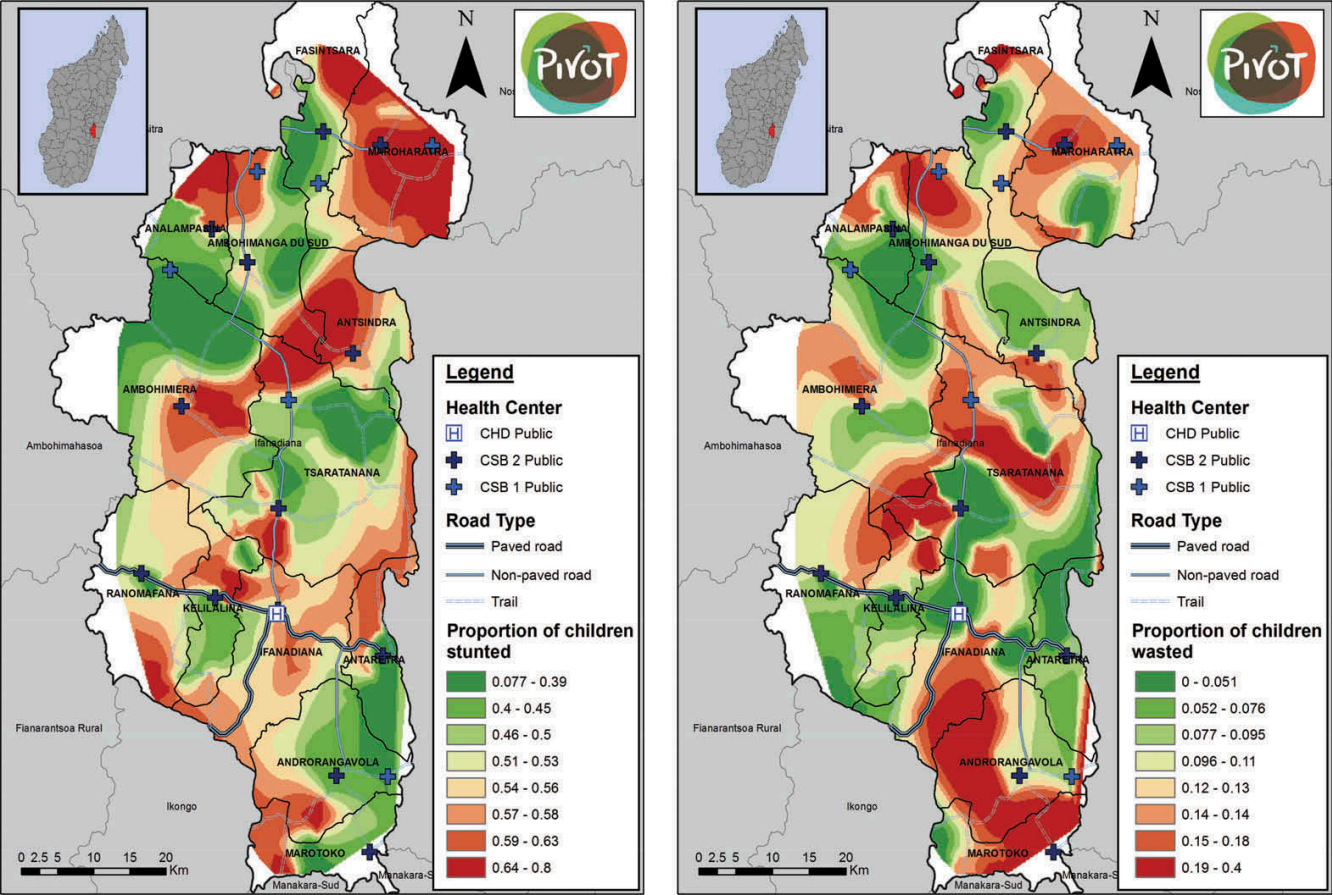
Geographic distribution of stunting (HAZ < −2) and wasting (WHZ < −2) in Ifanadiana district, by quantiles of each indicator.

The prevalence of stunting in the study group of children 6 months to 5 years of age was 50.5% and wasting was 10.6%. In the group of children 6–59 months of age, prevalences were similar (49.8% stunted and 10.8% wasted). Figure 1 shows the geographic distribution of stunting and wasting in the district, by quantiles of each indicator. The geographies of stunting and wasting have similarities, with less severe malnutrition along main roads and near most health centers, but they are not identical: most regions have a greater prevalence of either stunting or wasting, but few have a large prevalence of both. Descriptive statistics for the complete dataset for all variables analyzed are in Table 1, and show that the study population has high levels of ill health, poor sanitation, and relatively little access to preventive health services or education. Variables associated (p < 0.10) with stunting and wasting in univariate analyses are shown in Table 2, with survey-adjusted simple odds ratios displayed. Child sex was not associated with stunting or wasting; vaccination completeness was also not associated with either stunting or wasting. Final logistic regression models predicting stunting for groups A and B are shown in Table 2. For wasting, no paternal variables remained after backwards selection in group B, so only model A is shown.

For stunting (Model A1 in Table 2), child-level variables were the strongest predictors of those tested. The odds of stunting increased by 0.03 (OR = 1.03; 95% confidence interval, 95%CI 1.02–1.04) for each additional month of age. Very small birth size, as reported by each child’s mother, was associated with an increased odds ratio for stunting of 2.34 (95%CI 1.35–4.06), while smaller than average birth size was associated with increased odds of stunting, OR = 1.66 (95%CI 1.10–2.52). Maternal variables were also associated with stunting: each additional kilogram of mother’s weight decreased the odds of stunting by 0.07, OR = 0.93 (95%CI 0.90–0.96), while each additional centimeter of mother’s height decreased the odds of stunting by 0.04, OR = 0.96 (95%CI 0.92–0.99). In the subset of children with complete paternal data, group B, results were similar (Model B1 in Table 2), except that each additional centimeter of paternal height also decreased odds of stunting, OR = 0.95 (95%CI 0.92–0.98). Household wealth score was included in both stunting models as it slightly improved model fit for Model B1 and did not impact fit for Model A1, but its association with stunting was non-significant (p = 0.064) in both models.

For wasting (Model A2 in Table 2), predictors were somewhat different. Child-level variables remained important, but in contrast to stunting, each additional month of child’s age slightly decreased the odds of wasting, OR = 0.98 (95%CI 0.97–0.99). Very small birth size increased the odds of wasting, OR = 2.48 (95%CI 1.23–4.99), but the effect of smaller-than-average birth size was not statistically significant. Maternal variables were also important: a 1-point increase in maternal BMI was associated with lower odds of wasting, OR = 0.84 (95%CI 0.75–0.94). Maternal weight had a similar effect when it was substituted for BMI in the model, but maternal BMI was kept in the final model to improve model performance. Household wealth score was again not significantly associated with wasting, and was not included in the final model for wasting as it worsened model fit.

**Figure 2.**
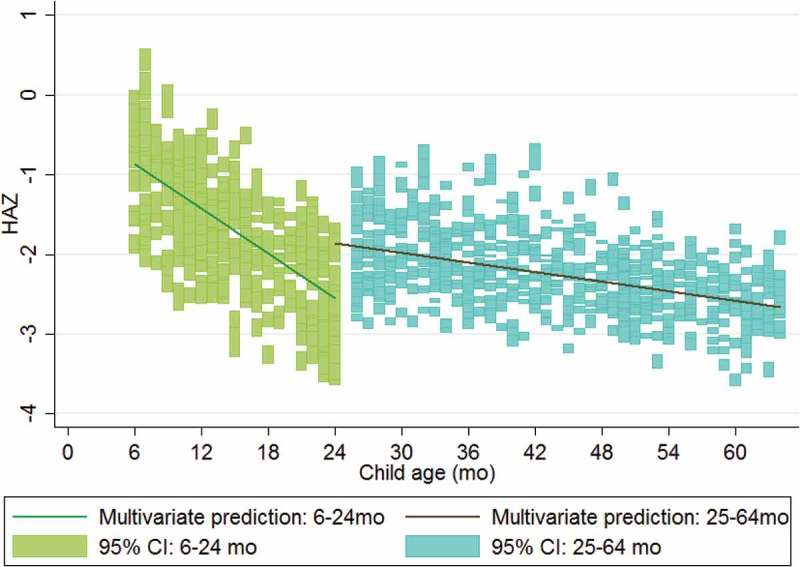
Multivariate model predictions for height-for-age z-score (HAZ) for children under and over 2 years of age.

**Figure 3.**
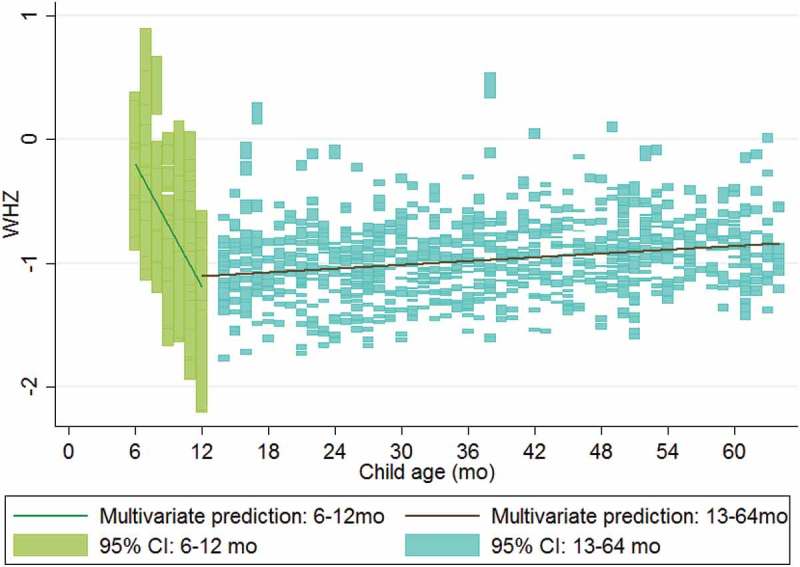
Multivariate model predictions for weight-for-height z-score (WHZ) for children under and over 1 year of age.

We present fully adjusted model predictions for HAZ under and over age 2 years in Figure 2, and we show predictions for WHZ under and over age 1 year in Figure 3. After adjusting for all significant predictors and household wealth, HAZ declines rapidly in the first 2 years of life, then more slowly afterward. WHZ declines even more rapidly within the first year of life, after which children recover toward the median, but do not reach the median before age 5.

## Discussion

Child malnutrition in low-income settings is an important driver of ill health and mortality. This is particularly the case in Madagascar where the stunting prevalence is the sixth highest in the world [35]. Understanding factors associated with both acute and chronic malnutrition in each setting can inform local implementation of interventions that aim to manage or prevent malnutrition in populations at risk. In this study, we used a cross-sectional survey conducted in Ifanadiana ahead of a health system strengthening intervention to shed light on the most important factors associated with malnutrition in the district’s population, and to establish baseline relationships that may change over the course of the intervention. The stunting and wasting prevalence levels found for Ifanadiana indicate an urgent public health situation according to WHO guidelines [36], and were similar to national estimates. We found that stunting and wasting had a heterogeneous geographical distribution in Ifanadiana, concentrated in remote areas far from roads and health centers. Both parental and child individual factors were important predictors of malnutrition in the district, but their effects differed for stunting and wasting. Faltering in both linear growth and weight gain occurred in very early life, as it does in international populations; however Ifanadiana’s children did not ‘catch up’ to international median growth by age 5 years.

Despite the wide variety of measured and tested variables, stunting is most associated with increasing child age, small birth size, low maternal height and weight, and low paternal height. Predictors of wasting were somewhat different, especially the protective effect of increased child age on wasting. Similar to stunting, the importance of small birth size was also evident. Higher maternal BMI had a strong protective effect, which appeared to be driven by greater maternal weight rather than lower maternal height. Taken together, these results suggest that of the factors measured, parental anthropometry and birth or gestational factors are most important in determining both linear growth faltering and wasting in Ifanadiana. These results are consistent with international literature showing that demographic factors, particularly child age, maternal weight, and birth size are important determinants of both stunting and wasting [13,14,17,19,21]. Paternal nutritional status is both less studied and less strongly predictive of malnutrition in international literature [17]; its importance in determining stunting may be a unique feature of our study, which included a large set of matched fathers, or of this population. This contrasts with previous studies in Madagascar which have attributed malnutrition to behavioral factors, poor maternal education, and male sex [25,27–29], and to international literature emphasizing parental education and behaviors [13,19] and male sex [13,17,21] as risk factors for malnutrition. The lack of association with vaccination and supplementation in our study provides evidence that these factors, which may mediate the relationships between parental education, work, and nutrition [18,20], are less important in this population. Environmental factors such as rural location predispose to malnutrition in this population, similar to international ones [13,17]. Interventions to address malnutrition in Madagascar, which have focused on parental education and supplemental feeding [26,27,30], may need to be augmented with earlier, household-level, and population-level interventions.

The influence of parental anthropometry on child nutritional status in this population may be genetic, epigenetic, environmental, or all three [37]. Although height and weight both have genetic components, growth faltering of this magnitude, this early in life, is very unlikely to be purely genetic [3]. It is likely that all members of households which experience food shortages, whether chronic or acute, experience growth limitation to varying degrees. This may also be true intergenerationally: small adults may have had childhood exposure to poor nutritional environments similar to those which later constrain their children’s growth [17]. Birth size’s importance in childhood growth faltering supports international evidence that some stunting is associated with gestational factors such as intrauterine growth restriction [4,13,19]. In particular, maternal stunting increases the risk of small-for-gestational age low birthweight in international populations [4], which may support a synergistic relationship between these predictors of child malnutrition in Ifanadiana.

Importantly in our study, current nutritional status was not significantly associated with recent illness, child vaccinations, or household water or sanitation, and its associations with wealth were equivocal and non-significant; child age and birth size may mediate these associations. In addition, the high prevalence of poverty, illness, and poor sanitation combined with the high rates of malnutrition (69% of children in the sample had been ill or injured in the past 4 weeks alone, and 99% of households had either an open pit or no facility for toileting) may obscure individual-level effects which have been noted in international literature [12–16] instead acting as a population-level environmental risk for poor growth [15].

As others have argued, early interventions are essential to prevent growth faltering, which happens very early in life [6]. This pattern appears evident in our study, in which HAZ falters more rapidly with age in the youngest age group (6–24 months) than in the older age group (25–64 months) or overall population from 0 to 5 years. Although stunting prevalence increases with age, the results support recent evidence that very early-life nutrition and exposures may be responsible for a disproportionate share of stunting [5,6,17,19]. Strengthening the district nutrition program, in which ANCs track detailed growth curves for each child, may be helpful in identifying children who are trending toward a diagnosis of stunting. Our results indicate that such intervention should happen early in the course of linear growth faltering, rather than waiting until a child is already stunted.

Also similar to global trends [6], children in our sample are most likely to be wasted between ages 6–12 months, and then become less vulnerable to wasting as their age increases, contributing to an overall protective effect of age on wasting. However, children in Ifanadiana who are wasted early in life do not recover to the median, as they do globally [6]. Intervention across the life course, as well as in the first 1000 days, is thus also indicated. To prevent wasting in children 6–12 months, and chronic malnutrition in children 6–24 months leading to stunting [4], complementary feeding during the transition from breast milk to solid foods could be emphasized or supported with food distribution in this population [9].

### Limitations

Some paternal data were missing, necessitating subgroup analysis of the children with paternal data (group B); however, univariate results and modeled predictors were consistent between groups A and B. Recall bias may be responsible for some of the observed association between birth size and stunting or wasting, as the relative birth size was a categorical comparison to peers reported by each child’s mother. Measurement error, particularly in self-reported illness variables, may lead to underestimation of illness, infection, and food availability as predictors; however the prevalence of illness was high in this population, making underreporting less likely to be responsible for its lack of association. Prevalence estimates in particular are very season-dependent, as these three factors also vary seasonally; however this does not affect the other conclusions of this cross-sectional study. Data on diet, food availability, and behavior were only collected for the youngest child in each family and were therefore not included in these analyses; they may additionally contribute to malnutrition in this population, and ongoing work addresses this possibility. Finally, these data are cross-sectional and results do not indicate causality, nor can they approximate stunting or wasting incidence.

## Conclusion

Child age, small birth size, and parental anthropometry are all important determinants of both stunting and wasting in Ifanadiana, consistent with international literature. The timing of growth faltering, in the first 1000 days, is also consistent with international populations; however median child growth in Ifanadiana does not ‘catch up’ to international median growth by age 5, as is typical for populations with growth faltering. Treatment for severe acute malnutrition according to national protocols and international guidelines should be scaled up in Ifanadiana, and monitored to assess effectiveness and population impact. More intensive case-finding for stunting, including the use of growth curves, could help identify children whose growth is faltering, particularly those below age 2 years. Further investigation of how and when complementary foods are introduced in children’s diets could help prevent both stunting and wasting. Further programming should address intergenerational transmission of growth failure, resource constraints, and gaps in maternal and perinatal care, which may be important determinants of child malnutrition in this district.

## Paper context

Child malnutrition is a leading cause of morbidity and mortality worldwide and is particularly severe in Madagascar, which has extreme poverty and a weak health system. Malnourished children’s growth falters during critical periods in early life and may not recover without effective treatment. We report factors predicting stunting and wasting and determine the timing of growth faltering in Ifanadiana district, Madagascar, to inform health system strengthening and malnutrition prevention and treatment initiatives.
